# Responding to COVID-19’s impact on supervised family time: The supportive virtual family time model

**DOI:** 10.1177/25161032211001078

**Published:** 2021-03-11

**Authors:** Laura Orlando, Ashley N. Rousson, Susan Barkan, Kristen Greenley, Alyssa Everitt, Emiko A. Tajima

**Affiliations:** 1 Partners for Our Children, USA; 2 University of Washington, USA

**Keywords:** COVID-19, family time, foster care, parent-caregiver relationships, parent-child supervised visits, visitation, virtual parenting

## Abstract

COVID-19 has put child wellbeing at risk, perhaps especially, for children and youth involved in the foster care system. For these children and youth, any stability they may have experienced since entering care has been disrupted and their contact with parents limited. A sudden shift to virtual only contact meant both parents and caregivers were in need of support to navigate these changes. This study reports on the rapid development and implementation of an eLearning and structured practice guide for visitation supervisors to help them facilitate virtual visits that promote parent-caregiver collaboration in support of the child. Over a four month period, April to August 2020, 140 people registered for the Supportive Virtual Family Time training. Of these participants, 101 (72%) completed a post-training survey which included an evaluation of the eLearning and assessment of the feasibility of implementing the model. Overall, participants were satisfied with the training content and delivery, agreed the training helped them develop new skills for interacting with parents and caregivers, and planned to start using the model right away. Given the ongoing and dynamic nature of the pandemic, there is likely continued need for virtual family time and training and support for supervisors who facilitate these interactions. It is critical these supports are timely, easily accessible, and include practice guidelines and resources that help practitioners facilitate and maintain youths’ critical attachments to their families. Given the potential benefits of supporting parent-caregiver collaboration, the model and accompanying materials address an overarching need in the field and remain relevant even after a return to in-person visitation.

## Introduction

Almost overnight, the COVID-19 pandemic put the wellbeing of children around the globe at risk, including children and youth involved in the foster care system. In the United States alone, this amounts to over 400,000 children (U.S. Department of Health and Human Services, 2019). For these children and youth, the pandemic has created upheaval in any stability and normalcy they may have experienced since entering care. The immediate shuttering of schools, the loss of normal routines, and social distancing risk impacting their future development and growth, sense of connection, and mental and physical health. Additionally, Black and Native American children are consistently overrepresented in the U.S. child welfare system (National Council of Juvenile and Family Court Judges, 2017). These same communities are disproportionately impacted by COVID-19 in the U.S. Data indicate that COVID-19 diagnosis and death are disproportionally higher among Black, Latinx, and Native American communities (Millett et al., 2020; Rodriguez-Diaz et al., 2020; Tai et al., 2020). These disproportionalities in risk for COVID-19 are the result of existing historical and structural inequities (Krishnan et al., 2020; Tai et al., 2020), that are also to blame for the disproportionate representation of these communities within the child welfare system (Drake et al., 2011). Effects of the pandemic alone are exacerbating mental health outcomes for these communities (Sneed et al., 2020), and the impacts for children who have already experienced the trauma of being placed in foster care, are likely greater (Collins & Baldiga, 2020).

Children living with out-of-home caregivers (which includes licensed foster parents and relatives—whether licensed or not) during this difficult time, need to feel safe and secure and have connection to family. The pandemic caused an immediate shift to virtual family time (supervised parent-child visits) for families around the world, as having in-person family time was no longer deemed a safe option. For parents, this situation is heartbreaking because they now can only hear or see their children over video, if at all. Additionally, caregivers are suddenly facing the challenge of having children in their care 24 hours a day, 7 days a week, while in many cases, also being responsible for supporting children’s schooling and connection to siblings.

Prior to COVID-19, the importance of maintaining consistent family time while children are in out-of-home placement was well-established (Hess, 1988; Nesmith, 2013). Consistent family time has shown direct benefits to children’s mental and emotional health, and improved outcomes in terms of length of stay in the system (Nesmith, 2013). Additionally, family time can help children in foster care to better manage the ambiguous loss which they often experience, a loss caused by the perception that the person is psychologically present, but physically absent (Lish, 2019). COVID-19, social distancing, and the inability to have in-person family time, can increase this sense of loss. These circumstances also put additional pressure on stressed child welfare systems that already offer little guidance or structure to parents and caregivers related to family time (Nesmith, 2013). There is little, if any, guidance specifically for virtual family time, which comes with its own unique set of challenges for all parties involved (Singer & Brodzinsky, 2020).

Both parents and caregivers need support to navigate this difficult and unprecedented time of global pandemic. In the last decade, there has been a movement toward birth parents and caregivers working together in a more collaborative fashion. However, this can be easier said than done, even without the added stress of a pandemic (Child Welfare Information Gateway, 2019). Healthy communication and collaboration between parents and caregivers has positive benefits for children and youth in care (Child Welfare Information Gateway, 2019). In some sense, children in foster care belong to two families and the relationship between these two families can be strained and complicated (Chateauneuf et al., 2018). These relationships have a direct impact on children, who may feel an attachment to both families and may be affected by tensions between parents and caregivers (Nesmith et al., 2017). In the virtual context, tensions in these relationships may be more visible to children as they witness both families interacting with one another more directly and frequently. Singer and Brodzinsky (2020) cite current evidence regarding virtual parent-child visitation suggesting virtual family time can support greater partnership between caseworkers, parents, and foster families, but underscore this will require appropriate training of all parties. Given the unprecedented nature of this time, we set about to develop a model to support parents, caregivers, and children in having positive virtual family time to help mitigate the impact of this potentially traumatic time. Additionally, given the potential benefits of supporting parent-caregiver collaboration, we suggest the model and accompanying materials address an overarching need in the field and thus remain relevant even after a return to in-person visitation.

## The present study

In this paper, we report on the rationale for and development of the Supportive Virtual Family Time (SVFT) model and training curriculum and present findings from our evaluation of the eLearning and survey of training participants. We describe the principles guiding the model, outline the components and structure of the SVFT model, and consider the promise it holds for supporting parent-caregiver collaboration. The findings from our survey of training participants provides learner feedback on the strengths and limitations of the curriculum and content, and offers participant input on the feasibility of implementing the model. Analyses include descriptive statistics regarding training participant characteristics, summary of responses to closed-ended post-training survey questions, and qualitative content analysis of open-ended survey items.

## Background

### Fundamental purpose of family time and skills needed

The Adoption and Child Welfare Act of 1980 (P.L. 96-272) placed such importance on family time that it requires the inclusion of regular family time in family preservation efforts (Haight et al., 2003). Despite there being clear evidence that family time is a crucial part of the family reunification process and it being a central mechanism for maintaining parent-child bonds and attachment, there is little guidance for family time supervisors on how to facilitate the quality family time that leads to positive interactions between parents, caregivers, and children (Nesmith, 2013). In fact, carefully planned and executed family time can provide support and insight into a number of critical areas such as: maintaining and improving family relationships, identifying areas in which the parent might need support in their parenting, providing data regarding parenting abilities, and encouraging the support and cooperation of caregivers (Kessler & Greene, 1999).

Parents, children, and caregivers need support and skills to manage emotions and behavior in and around family time. Nesmith (2013) identified five tenets related to best practice for family time:

1. **Preparation:** planning ahead for activities, what to bring, and what emotions might arise;
2. **Communication:** supporting open communication between parents, children, and caregivers;
3. **Emotions:** helping to connect how emotions not related to family time can impact family time;
4. **Connection:** intention related to the use of family time to enhance the parent-child connection; and
5. **Transition:** helping parents and children to transition in and out of family time (Nesmith, 2013).

These tenets hold true whether or not family time is in person or done virtually. Yet, we lack sufficient practice models to support this important but often missed opportunity. This paper describes a model to support virtual family time that was designed to fill this important gap. The virtual model builds on our work since 2014 developing and testing a family time support and education model for parents (Orlando, et al., 2019).

### Virtual communication: An opportunity to support parent-caregiver cooperation

In recent years, advances in technology have allowed for virtual interactions between parents and children when separated. Singer and Brodzinsky (2020) reviewed literature on virtual visitation and found it helpful in enabling children to maintain contact with their family. The use of such technology has been commonly available to parents who are incarcerated and parents who live long distances from children due to military service or divorce. One study on the use of remote technology for parents who were incarcerated showed benefits such as: increased frequency of regular and meaningful contact, reduction in separation anxiety for both parent and child, reduction of attachment disruptions, improved communication and interactions, more child-focused time, enhanced relationships, and families feeling more supported and less isolated (Spencer, 2011). In the field of child welfare, the use of video technology has also been on the rise. This use has largely supplemented in-person family time and is not used as the only form of contact between parents and children. The increased use of video technology has raised serious concerns about how this could impact bonding for these families, and the loss of in-person contact that is vital to keeping the connection in place could have long-term effects on reunification efforts (Pitzl, 2020).

Despite the unknown impact of virtual communication for families with children in out of home care, the COVID-19 virus has made virtual family time the only option for some. Due to the widespread nature of the virus and our lack of understanding about its spread and impact, social distancing and other safety measures were deemed a necessary and hopefully, temporary step, for controlling the virus. This resulted in the immediate shift to virtual only family time for thousands of families with children in out of home care. This shift to video technology for virtual family time is an opportunity to encourage better cooperation between parents and caregivers. As noted by the Birth and Foster Parent Partnership (2020), “Children and youth benefit by feeling safer and learning healthy communication skills when they see the foster parents/kinship caregivers and birth parents working together” (p. 5). One of the values of the SVFT model is the opportunity for children to see both parents and caregivers together and interacting in a collaborative way. This can help reduce the trauma of separation (Milner, April 2020) and support reunification (Ankersmit, 2016). Engaging in collaborative activities with the foster caregiver may also increase the engagement of birth parents (Birth and Foster Parent Partnership, 2020; Kemp et al., 2009). The goal of improving cooperation and collaborative relationships between parents and caregivers is a shift in the US foster care system which the federal Children’s Bureau (CB) supports. J. Milner, Associate Commissioner of the CB has noted how “critically important it is to move our foster care system in the direction of being more of a support to families and less of a substitute for them.” In an interview with Child Welfare Information Gateway, Milner underscores that “we’ve seen so much success when parents and resource families work together to give children what they need. This increases the chances that families can be reunited and allows children to thrive—even when it’s not possible for them to live under the same roof as their parents for some temporary period” (Milner, 2020). Neil and Copson (2020) point to opportunities that have opened up following the shift to virtual family time due to COVID-19, as foster caregivers who previously had no role in visitation “may now be helping children make video calls, giving carers and parents opportunities to talk and to work together more flexibly to find what works best for the child,” noting that “this in turn may help children feel that their birth family home and foster home are more integrated” (p. 2).

Building collaboration between caregivers and parents was a main driver in the development of a model to support family time. Given the unprecedented nature of current circumstances and the requirement for family time to be virtual, the following additional considerations were also taken into account in adapting the model:

1. **Attend to technology needs:** Do parents and caregivers have the necessary technology to support the meetings and virtual family time? If not, how can smartphones and tablets with data or Wi-Fi be provided quickly? What barriers to access might exist in rural communities? What platforms would provide reasonable security to users?
2. **Address safety concerns:** Some parents and caregivers have never met and caregivers might feel concerned about having parents “in their homes” and about what could be discussed. How can caregivers give some privacy to family time interactions, and at the same time support the parent-child connection?
3. **Support needs:** What support might parents and caregivers need in order to help create meaningful parent-child interactions and reduce trauma? How can both parents and children manage emotions related to not being able to see one another in person?
4. **Duration of family time:** How can family time supervisors and caseworkers help parents and caregivers make developmental adjustments to how long virtual family time lasts if for example, the court orders the family to visit for two hours twice per week with a toddler? How can realistic and developmentally appropriate online interactions be supported and negotiated? Are there other meaningful ways that this technology could be utilized to augment parent-child relationships such as being used to say good night or to share meals?

### Supportive virtual family time model

In response to the immediate need for support among parents, children, and caregivers, we rapidly developed the Supportive Virtual Family Time (SVFT) curriculum and training for providers. The training offers a format for facilitating virtual family time with parents, caregivers, and children in addition to providing a guide, resources, handouts, and videos aimed at improving the quality of virtual family time for children birth to eighteen years of age. Given our intention to help build the evidence base of best practices for virtual family time, we were thoughtful and strategic in terms of model development. We were careful to identify and outline the theoretical basis for the model, and clearly describe the curriculum components and training module. In our initial evaluation, we also attended to user experience of the eLearning training and perceptions of the feasibility of the model among child welfare staff and family time providers who took the eLearning.

**Figure 1.**
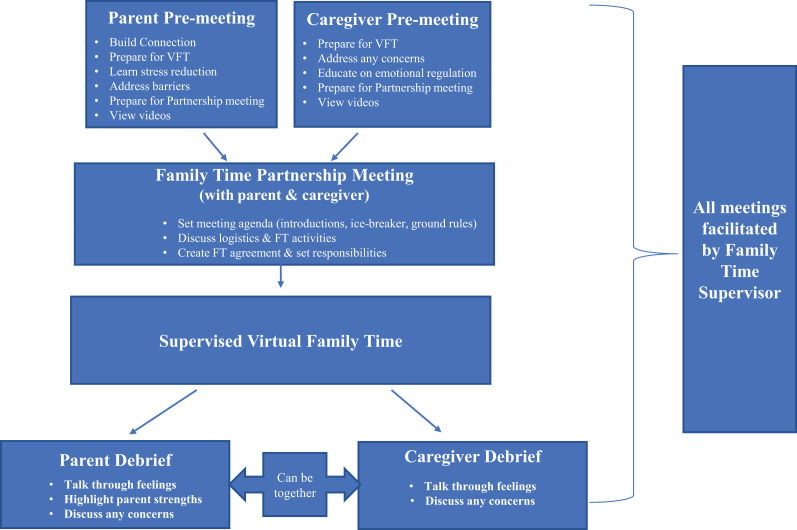
Supportive virtual family time model.

The SVFT model (Figure 1) builds upon the foundation and key principles of the *Strive* Supervised Visitation Program (Orlando, et al., 2019). *Strive* is a parent education and support program that assists parents in preparing for high quality visits with their children, and promotes child safety using a strengths-based, trauma-informed approach to help parents create a positive environment for nurturing their relationship with their child(ren) within the context of supervised family time. The SVFT model utilizes the key principles of *Strive* and adds a new critical element, the Family Time Partnership Meeting (FTPM). The FTPM component provides guidance for how to build a foundation for collaboration between parent(s) and caregivers in the context of virtual family time. It could also serve as an enduring model for supporting parents and caregivers in fostering a collaborative relationship early after a child has come into care.

#### Overview of the SVFT model

##### **SVFT model aims.** As part of the SVFT model, supervised family time providers are equipped with structured support, guidance, and training to

1. Connect with the caseworker to prepare for remote supervised family time that will, to some extent, include both parent and foster/relative caregiver(s).
2. Prepare the parent(s) for positive remote supervised family time with their children.
3. Prepare the caregiver(s) for how to support the child in their care in having positive remote supervised family time with their parent(s).
4. Hold a virtual FTPM between the parent(s) and caregiver(s) prior to supervised family time in order to plan and prepare for virtual family time by getting to know one another, setting expectations and agreements, and providing some structure for the remote family time.
5. Support the parent(s) and caregiver(s) in having positive and productive remote supervised family time and supervise this family time.
6. Create a plan to debrief one-on-one with the parent(s) and the caregiver(s) after the virtual family time meeting to celebrate successes and troubleshoot challenges.

### SVFT model components

1. A virtual meeting with the parent(s) before virtual family time takes place (30 minutes) with the following goals:
  - Establish a connection with the parent and understand unique challenges or concerns the parent may have
  - Prepare the parent for setting up a positive virtual family time with their child
  - Teach parent some stress reducing practices
  - Identify and address any barriers to virtual family time including access to technology and activities
  - Help parent create a routine for their family time
  - Prepare parent for their part in the FTPM
  - Provide encouragement and support by sharing a video of a positive virtual family time partnership meeting between a parent and a caregiver, and if time allows, also share a video of a positive virtual family time interaction
2. A virtual meeting with the caregiver(s) before virtual family time takes place (30 minutes) with the following goals:
  - Provide them with guidance on how to prepare the child for virtual family time and how they can support the child during this time
  - Address any safety or other concerns the caregiver might have
  - Educate caregiver about their role in modeling emotional regulation in the child
  - Prepare them for meeting with the parent in the FTPM, including sharing a video of a positive virtual FTPM between a parent and a caregiver, and if time allows, also share a video of a positive virtual family time interaction
3. A FTPM between the parent(s) and caregiver(s) before virtual family time takes place (1 hour) with the following goals:
  - Set an agenda for the meeting time which includes introductions/getting to know one another and respectful ground rules for their time together
  - Discuss logistics and activity ideas for virtual family time
  - Create a family time agreement and set responsibilities of both parties
4. A debriefing with the parent(s) and the caregiver(s) after the virtual family time takes place (15 minutes for each or 30 minutes together) with the following goals:
  - Provide a space and time to talk through feelings and have them validated
  - Discuss specific ways the parent stayed calm and positive with their child
  - If the meeting is held jointly, the caregiver can also be asked to provide positive feedback; the group can talk through any concerns

### SVFT model training and materials (free to access and use)

1. An hour long eLearning for supervised family time providers and caseworkers
2. A brief structured guide for supervised family time providers with suggested scripts and handouts for use in virtual meetings with caseworkers, parents, foster/relative caregivers, and during virtual supervised family time and debriefings
3. Video clip to accompany the curriculum featuring a parent ally, caregiver, and family time supervisor demonstrating how to work together to make remote family time a success
4. Suggested developmentally tailored activities
5. Resource lists for providers, parents, and caregivers (including Washington state-specific rules, guidelines, and tip sheets that can be tailored for use in other jurisdictions)

## Evaluation of eLearning and feasibility of SVFT model

### Method

#### Outreach and participants

We created a rapid deployment process in which both private and public agencies nationwide that work to serve children and families were emailed a link to the eLearning along with supporting materials about it. In late April 2020, information about the Supportive Virtual Family Time Model was posted on our organization’s website and social media. A description/announcement was distributed to over 700 stakeholders through an organizational newsletter and it was shared through several online websites, including the Washington State Alliance for Child Welfare Excellence Spotlight, and the University of Washington School of Social Work webpage. In addition, information was sent to national partners through NCFM@childwelfare.gov and the University of Albany as well as to the National Child Welfare Workforce Institute, the Associate Commissioner of the Children’s Bureau, Child Welfare League of America, The Center on the Developing Child at Harvard University, Families First.Org Resource Updates, Casey Family Models; the SVFT model is listed on the California Evidence Based Clearinghouse as a model with high relevance to child welfare, but one that is not able to be rated on their rating scale.

Given that caseworkers and supervised family time providers were the targets for the training and delivery, model developers worked with their state’s Department of Children, Youth, and Families who sent a message out to each of their regional Family Time Program Managers and placed a link to the model and eLearning on the resource page of their website. Model developers also delivered two presentations to a group of supervised family time providers as well as a webinar to a statewide court improvement group. It has been determined that this submission does not involve “human subjects” as defined by our Human Subjects Division. It does not require exempt status or Institutional Review Board review.

#### eLearning participant sample

Interested trainees registered to access the free, one hour SVFT eLearning and Model Materials through our online course catalog. The registration process captured basic demographic information of the trainees. Between April 28 and August 28 2020, 140 people registered for the training. This included private agency staff (20%), caregivers (9%), social work graduate students (14%), child welfare staff (45%) and other individuals with unspecified roles (12%). Of those who responded to the question about their time in social services, 44% reported 2 years or less, 21% reported between 2 and 5 years, and 20% reported 5 or more years. Eighty four percent of registrants were female, 13% were male, and 3% either selected “gender non-conforming,” “gender not listed,” or chose not to answer. Ten percent of registrants were less than 25 years of age, 31% were 25–34, 24% were 35–44, and, 30% were 45 years of age or older. Over half (53%) of registrants reported having a college or graduate degree, 7% had completed an associates degree, and 28% had completed high school or a GED program. Four percent of registrants identified as American Indian or Alaska Native, 9% as Asian, 14% as Black or African American, 11% as Hispanic or Latino, 2% as Native Hawaiian or other Pacific Islander, 54% as White, 4% as Multiracial, and 1% as another race/ethnicity. Seventy nine percent of registrants were from the state of Washington and 20% were from other US states.

#### Data collection

##### Post-training survey

Training participants were invited to take a voluntary post-training survey built using Research Electronic Data Capture (REDCap), an open source, web-based data collection application (Harris et al., 2009). The survey included 37 questions and took most responders about 15 minutes to complete. Survey questions asked about the following areas:

1. **Roles and Goals**—questions about participants’ roles, how they intend to use this training, readiness and plans to start using the model, and components completed
2. **Overall Assessment of the SVFT Model**—questions about the clarity and development of the model, trainer’s understanding of the subject matter, value of resources shared, ease of registration, participant assessment of new skills learned, and overall satisfaction
3. **Plans for Using the SVFT Model**—questions about the likelihood that participants will use the model components and their confidence in completing the meetings with parents and caregivers
4. **General Feedback**—questions about aspects of the model that were the most helpful or needed to be changed or improved, or that might be difficult to implement.
5. **Participant Demographics**—including gender identity, age range, level of education and race or ethnicity
6. **Qualitative Feedback**—open-ended questions regarding readiness to implement the model, aspects of the training that were most helpful, aspects that need to be changed or improved, and aspects of the model that may be difficult to implement

#### Data analysis

Basic descriptive statistics, including frequency distributions and bar charts, were used to summarize and analyze quantitative data derived from the surveys using tools available in REDCap. Qualitative responses to open-ended questions were downloaded from REDCap and summarized categorically by the project team. The project team, which consisted of five individuals, also conducted qualitative analysis of four brief, open-ended questions using content analysis and inductive coding (Vaismoradi et al., 2013). Independent coders reviewed participant answers to each of the four questions and coded each response with the goal of describing and summarizing the content of these answers (Vaismoradi et al., 2013). In pairs, the independent coders discussed their codes to resolve any discrepancies and come to agreement on the qualitative theme and choose illustrative quotes. In our analysis of the qualitative data we coded each written comment. In many instances, comments yielded multiple codes, therefore, in the write-up of the qualitative findings, the number of respondents may add up to more than 100%, as some participants made multiple points in each written comment.

#### Results

Of the 140 people who completed the SVFT training, 101 (72%) completed the post-training survey. Of those who completed the survey, 60% were family time supervisors. The remaining participants were graduate students doing practicum work in child welfare (11%), child welfare caseworkers (8%), child welfare supervisors or administrators (6%), and family time provider administrators (5%). The remaining 10% specified a variety of roles including foster parent, foster care or family support worker, child placement case manager, and intern. Participant reports of experience in their current role ranged from less than one year (31%) to 5 or more years (16%), with 22% reporting 1–2 years of experience and 31% with 2–5 years of experience.

##### Purpose and completion of supplemental eLearning elements

Most survey participants (89%) reported that they completed the training in order to use the content to facilitate virtual family time and 75% said they planned to use the model. All of the participants who completed the survey viewed the eLearning training video and 92% reviewed the structured guide for conducting the model’s pre-meetings, partnership, and debriefing meetings with parents and caregivers.

##### Demographics of those who completed the post-training survey

Seventy nine percent of survey participants noted their gender as female, 14% as male, 1% transgender, 4% indicated they preferred not to answer this question, and 2% were missing. Participants were from the following age categories: 18–29 years (28%), 30–39 years (18%), 40–49 years (22%), 50–59 years (15%), and 60 years or older (8%). Eight percent stated they preferred not to answer the question and 2% were missing. Over half (60%) of the participants reported having a college (32%) or graduate degree (27%). Twenty-seven percent had completed some college or trade school and 8% reported their level of education as high school or less. A small percentage (5%) preferred to not answer this question and 2% were missing.

When asked about their race or ethnicity, participants had an opportunity to select multiple response categories. Six percent identified as American Indian or Alaska Native, 10% as Asian, 23% as Black or African American, 11% as Hispanic, Latinx, or Spanish, and 51% as White. Eight percent said they preferred not answer the question and 2% were missing. It is worth noting that the majority of survey respondents (76%) were from the state of Washington, where Census 2010 reports that 77% of the population identified as White. Eighteen percent of respondents were from other states which included small clusters of participants from the Washington DC area, California, and Kansas. There were also participants from Colorado and Nebraska.

##### Participant experience and satisfaction with the eLearning

The SVFT model was very well received by those who completed the post-training survey (Table 1). All of the participants agreed that the learning objectives for the training were clearly identified, 99% agreed that the content was logical, coherent, and well developed and that the trainers displayed a clear understanding of the subject matter. The eLearning approach was also well-received, with 97% agreeing that it was conducive to learning. Just over 90% of survey participants found it easy to register for the eLearning. Overall satisfaction with the training was high, with 95% saying they were satisfied. We found that 94% agreed that they had developed new skills for interacting with parents and caregivers.

**Table 1.** User satisfaction with the eLearning.

Scaling Question	Agree		Uncertain		Disagree	
	n	%	n	%	n	%
The learning objectives were clearly identified	101	100	—	—	—	—
The eLearning approach was conducive to my learning	98	97	3	3	—	—
The content presented was logical, coherent, and well developed	98	99	1	1	—	—
The trainers displayed a clear understanding of the subject matter	100	99	1	1	—	—
The resources and links included in the model were helpful	97	97	3	3	—	—
It was easy to register and navigate this online course	92	91	4	4	5	5
As a result of the training, I have developed new skills for interacting with parents and caregivers	95	94	5	5	1	1
Overall, I am satisfied with this training	94	95	4	4	1	1

*Note.* N = 101.

Additionally, participants were asked “What aspects of the Supportive Virtual Family Time training were most helpful for you?” A majority (87%) of the 101 participants responded to this question, resulting in 88 respondents, whose comments yielded 99 coded responses. The majority (95%) of these coded responses indicated a specific aspect of the training that was most helpful. The most common response (42% of the 99 coded responses) indicated the materials and examples provided were most helpful. As one participant noted, “*Watching the example of the meeting between foster parent and biological dad was helpful to get an idea of my role in it and how that would look.*” Another stated, “*What was most helpful was the resources and scripts you can use during supportive virtual family time.*” The next most helpful aspect of the training (21%) related to the model itself and how each of the meetings had a purpose and structure. For example, “*Having a virtual meeting with parents and caregivers before starting visits with kids so caregivers and parents can get to know each other.*” Twenty percent of respondents felt the supportive nature of the model was helpful. Such as the participant who stated the helpful nature of, “*knowing how to stay positive even with negatives from parent or caregiver.*” Twelve percent indicated that the overall model or *all* aspects of the model were helpful. A small percentage of participants (2%) felt that the training was informative, but not applicable to their role. Another small percentage (3%) indicated they did not find the training helpful.

Participants were also asked what aspects of the Supportive Virtual Family Time training need to be changed or improved. Out of 81 responses, 12 responded “n/a” or not applicable, and were not analyzed. There were 76 codes applied to the remaining 69 total responses. Of these 76 codes, 30 (39%) were coded training is great as is/nothing to improve. Twenty-nine (38%) of the 76 coded comments provided suggestions for improving the content or delivery of the training and fit into two broad themes, more video examples, and presentation strategies.

Those asking for more video examples often cited the need for more diversity in scenarios addressed in the videos including the need for more examples of handling challenging visit scenarios, and increasing the diversity of family demographics represented. For example, one participant stated, “*I think we need to see an example of the caregiver not being as forthcoming with information or the facilitator having to actively keep the conversation on track*,” while another suggested, “*more demographic/racial representation*,” would be helpful.

In terms of presentation strategies, participants provided comments such as, “I felt the 50 minute training video could have provided more focused summary of accompanying guideline document,” and “I believe the videos were on the lengthier side.” The remaining 17 coded comments (22%) focused more on suggestions for improving the content of the model itself. Four participants (5%) expressed doubts about the feasibility of some of the model components such as the number of meetings, or specific meetings such as the parent/caregiver meeting. Additionally, 13 participants (17%) made specific suggestions regarding adding or changing components to the model such as more inclusion of caregivers in facilitation, the addition of sibling visits, more training for caregivers or supervisors, or changes to the model meetings. Two participants (3%) suggested including content on assisting with technology, and two suggested that overall, some of the content lacked clarity.

##### Participant implementation readiness and perceptions of model feasibility

Additional qualitative responses captured information about participants’ plans and readiness to implement the model, as well as more details about perceptions of initial feasibility of the model. Specifically, participants were asked whether they were ready to start using the model. Of the 100 responses to this question, 65 (65%) indicated “Yes,” they plan to start using the model right away. Another 10 (10%) said “Yes” they plan to use the model but indicated they have further planning to do before starting. Two (2%) responded “No,” and indicated they would like to use the model but were not ready to start. Six (6%) said “No,” stating they were interested in the training but have no plans to start using the model at this time. One had no response and 17 (17%) responded “Other.”

Of those who answered “Other,” 17 shared qualitative comments, which were analyzed by the project team. The responses for the 17 participants yielded 21 coded comments. Eight participants indicated that they were currently doing some form of virtual family time and seven said they had done the training to further their education on the topic. For instance, “*The agency has been doing virtual visits for about 7 weeks. This has been very helpful. I have been teleworking and may be on my way back to doing visits with families, which may continue to be virtual.*” Another participant stated, “*We are doing most of these things already in family time we will add this curriculum/strategies to what were currently doing to improve and make family time more successful.*” In addition, two participants used the “Other” response to clarify that they were not a family time supervisor. For example, “*We provide support for foster homes so would need to partner with child placing agencies.*”

Four participants indicated that they were unsure about their readiness to start using the model. Further, participants were asked, “Are there specific components of the Supportive Virtual Family Time model that you think will be difficult to implement?” A small number of participants (n = 21) shared their thoughtful reflections on the possible challenges with delivering the model. The most common concern, expressed by 8 participants, related to possible resistance on the part of caregivers or parents, particularly resistance to the partnership meeting. As one participant wrote, “*I think it will be hard to have a parent/caregiver pre-meeting. Some caregivers and parents are just not comfortable with meeting one another.*”

Several participants pointed to the number of meetings as representing a potential challenge to implementation. One wrote, “*All of the different meetings [will be difficult to implement].*” Parent-related issues were mentioned as another possible obstacle. For example, one participant noted, “*I expect some parents will be unhappy continuing to have virtual visits regardless of the necessity to do so to protect safety*.” Technology and the logistics of the implementation were identified as a possible barrier as well: “*Technology can increase our access, especially during this time, but it can also be a barrier for some folks, and I expect that there will be some difficulty implementing this model unilaterally.*” One respondent suggested that the stress reduction exercises might be hard to implement. In their words, “*With some families it will be difficult to do some of the calming exercises*.” Two responses which were found to be not applicable were not analyzed.

## Discussion

The aim of this work was to develop and quickly implement an eLearning for visitation supervisors and caseworkers in the foster care system to support them as they had to rapidly convert from in-person to virtual visits due to COVID-19. The eLearning provided a model for how to support parents and caregivers during this challenging and unprecedented shift as it could be the first time they are meeting each other and collaborating on behalf of the child(ren). Given these factors, we assumed support would be needed to make this transition go as smoothly as possible for all involved. Using a structured survey with quantitative and qualitative questions, researchers captured participant experience and satisfaction with the training as well as readiness to implement the model and initial perceptions of the model’s feasibility. Participants showed high levels of engagement and nearly universal satisfaction with the eLearning training. In qualitative responses, participants indicated that the specific practice examples and the supportive nature of the content were most helpful. These findings suggest model content and training approach for this eLearning address a key gap in education and support needs among professionals engaged in supporting families to have quality family time. The importance of quality family time for parent-child connection and child wellbeing, as well as the dearth of practical support in the field for this, are documented in the research literature (Nesmith, 2013; Singer & Brodzinsky, 2020).

Further, high levels of participant satisfaction and perceived knowledge and skill acquisition related to facilitating family time are promising indicators of whether transfer of learning might occur (Mansour et al., 2017). The vast majority of trainees (95%) were satisfied with the training and 94% stated they developed new skills for interacting with parents and caregivers as a result of the training. Additionally, 91% felt that the eLearning was easy to register for and navigate, which indicates this is a promising format for future eLearning trainings.

Additionally, we asked participants about their readiness and intention to use the model following the training. Overall, 87% of those who took the post-training survey provided information about their readiness and intention to use the model. Of these responses, 88% indicated they were currently ready, or with some additional planning would be ready to implement, and intended to do so. In qualitative comments in follow-up to this question, a few participants questioned the feasibility of certain aspects of the model. In particular, participants voicing concern indicated they worried about resistance on the part of parents and caregivers to this sort of collaborative approach. We were not surprised to see participant comments about possible resistance on the part of parents or caregivers. Collaboration between parents and caregivers is not routinely facilitated by caseworkers, and strained relationships between parents and caregivers are well documented (Chateauneuf et al., 2018; Nesmith et al., 2017). Tensions may stem from inadequate communication between caseworkers, parents and caregivers, anxiety about the visit, feelings of anger, or competitiveness on the part of parent, and caregiver perceptions that they are regarded as the “enemy” or that visits with parents negatively affect the children (Chateauneuf et al., 2018; Nesmith, 2013; Nesmith et al., 2017). Nesmith et al. (2017) found that caregivers were reluctant to collaborate with parents until they felt greater empathy for them, while parents felt negatively toward caregivers until their anxiety and feelings of vulnerability and powerlessness were eased. Although resistance to collaboration on the part of parents or caregivers may be a challenge to the SVFT model, there are significant benefits to positive and collaborative relationships between parents and caregivers and we encourage the use of family time partnership meetings between parents and caregivers to support this goal. While identifying potential barriers to implementation is critically important, it is notable that feasibility concerns were expressed by only 10% of post-training survey respondents.

Child welfare research and policy emphasize the importance of frequent and high quality family time to maintain parent-child connections that are essential for child wellbeing and predictive of successful outcomes for child welfare involved families (Child Welfare Information Gateway, 2019; Milner, 2020; Nesmith, 2013). Despite this, there is evidence that front-line service providers lack the training and support to facilitate supportive family time and strengthen these important relationships during family time (Nesmith, 2013). While some may caution against too much reliance on virtual contact between parents and children in out-of-home care (Pitzl, 2020), the health and safety risks posed by the current COVID-19 pandemic have made virtual visits, at least for a time, the only option for families and service providers. Even with a shift back to in-person family time, it is likely that many months of living nearly entirely “virtual lives” will have long-term implications for the use of technology in personal and professional interactions, both in the child welfare realm and elsewhere. As Singer and Brodzinsky (2020) have noted, “With appropriate training, virtual visits can be used as a valuable adjunct to face-to-face visits, offering more contact between birth parents and their children, supporting the enhancement of parenting skills and fostering stronger and healthier parent-child bonds” (p. 168).

The known gaps in support and training combined with high levels of engagement and satisfaction with the SVFT training, and strong indication of plans and intent to implement suggest this model, training, and eLearning delivery format hold promise for the field. In particular, this approach provides an easily accessible, free, evidence-informed and well-structured training for direct service providers. Additionally, the content aims to enhance coordination and relationship building between parents and caregivers, which holds promise for child wellbeing and reunification outcomes (Ankersmit, 2016; Haight et al., 2003; Neil et al., 2020; Nesmith, 2013). The free, online training format and curriculum materials increase accessibility to the model and training support for providers, which may increase equal access to such tools. The reliance on at least some virtual family time in the future could also increase equity within the system given the known barriers to visitation such as lack of transportation, far away placements, and conflicting demands of work and family time schedules. Further, the FTPM component of the model lends itself to both virtual and in-person family time, thus, making it an option for families who are utilizing in-person, virtual, or hybrid family time approaches now, and in the future.

Future plans to evaluate the utility of the SVFT model include asking participants who planned to use the model to complete follow-up surveys assessing whether or not they did, in fact, use the model, what components they used, how they experienced the implementation process, and what barriers they identified. An added and important dimension for future study would be to collect data from parents and caregivers alike to see how they experience the model and explore whether it played a helpful role in creating a more collaborative and supportive virtual family time experience. It will be important to capture the race/ethnicity of parents and caregivers and examine equity in access to the model and whether or not the model met their needs in terms of improving the quality of the family time and their ability to collaborate to support the children’s wellbeing. This additional data collection could help to inform modifications to the eLearning and model, such as ways to increase the feasibility of the meetings and adding new video scenarios.

### Limitations

This study’s contributions should be evaluated in light of some limitations. First, participants completed surveys post-training only. Results related to knowledge and skill acquisition could have been strengthened by a pre-post survey design. Additionally, while we gauged participant satisfaction and confidence in using the model and materials, we did not assess whether they actually used the content nor what barriers might have emerged for them as they did. Only with this type of follow-up will we know the extent to which trainees were able to transfer their learning to the work environment, if some parts of the model were more challenging to implement than others, and what actual benefits of fostering a collaborative relationship between parents and caregivers resulted from using the SVFT model. Also, we spoke only to service providers. In order to evaluate the full utility of the model in practice, it is critical to hear directly from parents and caregivers engaged in this approach about their experiences. In particular, it would be important to know whether the collaborative meetings were helpful in improving virtual family time, and fostering relationships between parents and caregivers.

## Conclusion

As the COVID-19 pandemic drags on, limiting in-person contact, there will likely be a continued need for virtual family time. Virtual family time could also become an enduring option for supplementing and/or expanding parent and child contact during out-of-home placement. Virtual family time can help maintain bonds, especially when social distancing is needed and/or parents live distant from where their children are placed. Virtual Family Time Partnership meetings could provide an opportunity for parents and caregivers to meet, to set up roles and expectations related to family time, as well as provide a meaningful opportunity to build cooperation regardless of whether the family time happens virtually or in-person. Given the high level of reported skill acquisition and satisfaction with the training content and delivery mechanism, providing training via eLearning with resources to facilitate these meetings virtually with parents and caregivers seems like a feasible way to accomplish this goal.
